# Melittin Inhibition and Eradication Activity for Resistant Polymicrobial Biofilm Isolated from a Dairy Industry after Disinfection

**DOI:** 10.1155/2019/4012394

**Published:** 2019-01-15

**Authors:** Emilia Galdiero, Antonietta Siciliano, Renato Gesuele, Valeria Di Onofrio, Annarita Falanga, Angela Maione, Renato Liguori, Giovanni Libralato, Marco Guida

**Affiliations:** ^1^Department of Biology, University of Naples Federico II, Via Cinthia, 80126 Naples, Italy; ^2^Department of Sciences and Technologies, University of Naples “Parthenope”, Business District, Block C4, Naples 80143, Italy; ^3^Department of Agricultural Science, University of Naples Federico II, Via Università 100, 80055 Portici, Napoli, Italy; ^4^Department of Movement and Wellbeing Sciences, University of Naples “Parthenope”, Via Medina 40, Naples 80133, Italy

## Abstract

The emerging concern about the increase of antibiotic resistance and associated biofilm has encouraged scientists to look for alternative antibiotics such as antimicrobial peptides (AMPs). This study evaluated the ability of melittin to act as an antibacterial biofilm inhibitor and biofilm remover considering isolates from dairy industry. Minimum inhibitory concentrations (MICs), minimum bactericidal concentrations (MBCs), minimum biofilm inhibitory concentrations (MBICs), and biofilm removal activities were studied in polymicrobial biofilms produced from isolates. MIC and MBC were set at 1–3 *µ*g/mL and 25–50 *µ*g/mL for Gram-positive and Gram-negative bacteria, respectively. Results demonstrated a good MBIC reaching 85% inhibition ability and a good activity and better penetration in deeper layers against the mixed preformed biofilm, thereby increasing its activity against all isolates also at the lowest tested concentrations. Melittin showed interesting characteristics suggesting its potential to act as an antimicrobial agent for polymicrobial biofilm from dairy industry even in environmental isolates.

## 1. Introduction

There is a pressing need to develop new antimicrobial agents active against bacteria and able to avoid drug resistance in order to face the huge public health problem correlated to the increasing emergence of multidrug-resistant bacteria [1, 2].

Antimicrobial peptides (AMPs) are an attractive solution as therapeutic agents, for their capability to kill a broad range of bacteria (including multidrug-resistant strains), fungi, and viruses and reducing the development of drug resistance [3–6]. Natural antimicrobial peptides (i.e., >750 compounds) are considered alternative agents with new active mechanisms of action and a wide diversity of active principles [7]. Bee venom (BV) is a complex mixture of peptides, enzymes, and biogenic amines possessing several pharmaceutical properties, whose main peptidic components are melittin and apamin. Melittin is a water-soluble toxic 26 amino acid peptide. It constitutes about 40–50% of the dry weight of BV being the most active toxin. It has antibacterial, antiviral, anti-inflammatory, and antifibrotic properties [8–11], developing a rapid cytolytic action and destabilizing membranes of different cell types [12–15]. Melittin presents a strong binding affinity with membranes due to its amphiphilicity, which is correlated to the asymmetric distribution of polar and nonpolar side chains. This ability provides its therapeutic potential for various bacterial and viral diseases, as well as inflammatory events and cancers.

An important public health concern is represented by antimicrobial resistance in bacteria potentially transmitted by foods, and studies have been recently devoted to unravelling possible factors influencing the emergence of antimicrobial-resistant bacteria in the food chain. Globally, foodborne diseases are now among the most widespread public health problems due to contamination during production, collection, transportation, processing, and storage [16–18].

The ability of microorganisms to resist to adverse factors and colonize the environment is further enhanced by biofilm production. Biofilms are sessile bacterial communities attached to a substrate and embedded in a self-produced extracellular polymeric matrix. In this polymeric matrix, cells exhibit a distinct phenotype, metabolism, physiology, and gene expression compared to the planktonic ones [19].

Bacterial biofilms are a source of constant contamination of the product and thus represent a risk in the food industry. In particular, all places where food comes in contact with food are subject to this problem, such as stainless steel pipes, utensils, tables [20–22]. Furthermore, in the food industry, the common disinfecting agents used for food facilities could have negative effects, such as the toxicity of residues, the promotion of microorganisms resistance [23], and a decreasing susceptibility of biofilms to them.

In recent years, lot of efforts has been made to find new antibiotics to which bacteria forming biofilm cannot develop resistance. Antimicrobial peptides, isolated from natural sources, including mammals, insects, and plants, have emerged as good candidates [24]. Especially, some venoms were reported to have antibiofilm activity [25].

To guarantee the optimal level of equipment hygiene in the dairy industry, it is necessary not only to kill bacteria and to inhibit their capacity to form biofilms but also to remove effectively from surface-attached bacteria.

This study aimed at investigating new antimicrobial molecules from natural compounds having effective structures such as melittin. This study was carried out to (i) evaluate the ability of strains isolated from dairy industry to form biofilms on hydrophobic surfaces at 37°C; (ii) determine the minimum inhibitory concentrations (MICs), minimum bactericidal concentrations (MBCs), and minimum biofilm inhibitory concentrations (MBICs) of melittin on dairy industry isolates; and (iii) test the ability of melittin to inhibit and to eradicate their biofilms formation.

## 2. Materials and Methods

### 2.1. Melittin Production

Melittin was obtained using standard solid-phase-9-fluorenylmethoxycarbonyl (Fmoc) method a on a Rink amide MBHA resin (scale of 50 mmol) as previously reported [26].

### 2.2. Collection of Samples

Samples were collected in a dairy plant tank used for mozzarella cheese production located in Campania region (Italy) after cleaning procedures. For surface samples from post-disinfection tub, sterile swabs moistened in saline (0.85%) with peptone (0.1%) and sodium thiosulfate (0.01%) were used in the defined area of 100 cm^2^ following the ISO 18593 : 2004 [27]. From collected samples, isolation of typical colonies was performed according to ISO 4833-1 : 2013, ISO/TS 11059 : 2009, ISO 6888–1 : 2004, ISO 4832 : 2006, and ISO 16649–2 : 2001 [27–31]. Isolates were maintained at −18°C until DNA extraction. Samples selection of the strains to be used in the experiments was performed after biofilm production assay. From the total of isolates, four were selected as biofilm producers. The capital letters before strains indicated the isolation origins, so R1-R2 mildly forming biofilm, R3 strongly forming biofilm, and R4 weakly forming biofilm strains were selected for our study and molecularly identified.

### 2.3. Molecular Characterization

One colony from the selective medium containing our isolates was reconstituted with 50 *μ*L Milli-Q Type 1 Ultrapure Water and DNA extracted through denaturation at 96°C for 10 minutes and then centrifuged at 10,000 rpm for 10 min at 4°C [32]. The supernatant was recovered and used for polymerase chain reaction. PCRs were carried out in a Techne Prime Thermal Cycler. The bacterial DNA was amplified using universal PCR primers, complementary to V3 and V6 regions (16S rDNA) [33]. The selected oligos were V3_F (5'CCAGACTCCTACGGGAGGCAG-3') and V6_R (5'-TCGATGCAACGCGAAGAA-3'). A typical 25 *μ*l PCR reaction contained 50 *μ*M of each invA primer, 0.2 *μ*M of each dNTP (VWR Chemicals), PCR Key Buffer Triton Free (Tris-HCl, pH 8.5, KCl, 15 mM MgCl_2_ (VWR Chemicals)), 1 *μ* VWR Taq DNA polymerase (VWR Chemicals), and 0.5 *μ*L sample DNA. The incubation conditions were 95°C for 2 min (initial denaturation), followed by 30 cycles of 95°C for 30 s, 55°C for 30 s, and 72°C for 45 s. A final extension of 72°C for 5 minutes was employed. The amplified products were visualized and analyzed for size on agarose gel (1.5%), stained with GelRed (nucleic acid gel stain-BIOTIUM) using DNA 100 bp ladder as a reference. DNA sequencing was performed by Bio-Fab research s.r.l. (Italy). Sequences obtained were compared and aligned to those available in the NCBI sequence database.

### 2.4. Biofilm Production

Strains were cultured in 5 mL tryptone soya broth (TSB) medium (Difco) for 24 h at 37°C and were suspended at 10^7^ cells/mL in fresh TSB. Then, 100 *µ*l of the suspension was added into individual wells of polystyrene 96-well plates and incubated at 37°C for 24 h to allow the develop of the biofilm. The total biomass of the biofilm was analyzed using the Crystal violet (CV) staining method [34], as described elsewhere [3, 35]. Briefly, after the incubation, wells were washed with 300 *µ*L of PBS to remove unattached bacteria and air-dried at 60°C for 60 min. Then, 150 *µ*L of CV (0.2% p/v) was added to each well and incubated for 15 min. After washing the wells with deionized water, excess stain was gently rinsed off with tap water. CV bound to the biofilm was detached using 150 *µ*L of 30% v/v acetic acid for 30 min at room temperature, and the absorbance at 570 nm was detected with a spectrophotometer (DR5000, HACH).

### 2.5. MIC and MBC Determination

MICs of melittin, on our isolates, were determined with a microbroth dilution technique as described by the Clinical and Laboratory Standards Institute [36] (CLSI, 2006 M7-A6) using TSB. Melittin was tested at concentrations ranged from 5 to 100 *µ*g/ml. Plates were incubated at 37°C for 24 h and examined. Wells without the test molecule served as control. The MIC was defined as the lowest concentration of peptide that completely inhibited visible growth analyzed at 590 nm using a microplate reader (Synergy H4 BioTek). MBCs were determined at the end of the incubation period by subculturing 10 *µ*l samples from each well, demonstrating no visible growth and plated onto tryptic soy agar (TSA, Difco Laboratories) medium plates. Resultant colonies were counted after an overnight incubation at 37°C. The MBC was defined as the lowest concentration of antimicrobials that killed at least 99.9% of the initial inoculums [37]. All tests were performed in triplicate. The MBC/MIC ratio was calculated to determine whether the substance had a bacteriostatic (MBC/MIC ≥ 4) or bactericidal (MBC/MIC < 4) activity [38].

### 2.6. Inhibition and Eradication of Biofilm Formation Test

*Pseudomonas aeruginosa* strain RMR101 (R1), *Staphylococcus haemolyticus* strain M2 (R2), *Klebsiella pneumoniae* strain 459 (R3), and *Aeromonas caviae* strain 8LM (R4) were grown overnight at 37°C in TBS, washed twice in PBS, and resuspended to obtain a suspension equivalent to 1 × 10^5^ cells/ml (OD600) when we tested each one biofilms and 2,5 × 10^4^ cells/ml each when we tested all four biofilms together. 100 *μ*l of each inoculum was dispensed into wells of 96-well microtiter plates.

To prevent cells adherence and to value the capacity to form biofilms at the intermediate stage (24 h biofilms), the plates were incubated at 37°C for 24 h with melittin at concentration of 5–100 *µ*g/ml, while to eradicate preformed biofilm at the maturation stage (48 h biofilms), the plates were incubated for 48 h, the medium was renewed after 24 h, and melittin at the same concentrations was added at the last 24 h.

Biofilms formed by bacteria that did not undergo any treatment with melittin were used as controls for comparison with the means of the treatments.

The effect of melittin on biofilm inhibition and eradication was quantified by using the XTT assay that analyzed the density of the adhered cells, measuring the relative metabolic activity using the XTT (2,3-bis (2-methoxy-4-nitro-5-sulfophenyl)-5-(phenylamino) carbonyl)-2H-tetrazolium hydroxide) colorimetric assay kit (Sigma) following manufacturer's instructions as descripted elsewhere [39].

Scanning electron microscopy (SEM) was used to visualize the effect of melittin on the biofilm eradication. Preparation of samples for SEM was performed as previously described [40].

Data were assessed considering the analysis of variance (ANOVA) and Tukey's test to check any difference among the groups after lognormal transformation of concentration data using Microsoft® Excel 2013/XLSTAT©-Pro (version 7.2, Addinsoft, Inc., Brooklyn, NY, USA). When ANOVA revealed significant differences among treatments, post hoc tests were carried on with Dunnett's method testing the pairwise difference between each treatment and the control. Parametric methods were considered for points' estimation. Pearson correlation analysis was used to compare the inhibition and eradication of single as well as polymicrobial biofilms (PMBs) (*p* < 0.05).

## 3. Results and Discussion

**Figure 1:**
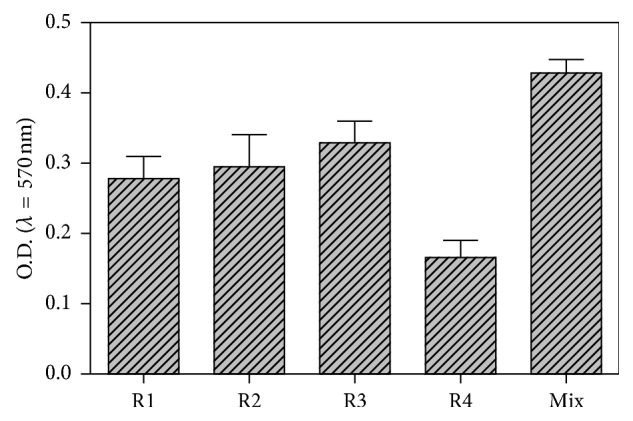
Total biomass of monomicrobial and polymicrobial biofilms of the diary isolates adhered on polystyrene. Negative (OD ≤ ODc), weak (ODc ≤ OD ≤ 2.ODc), moderate (2.ODc < OD ≤ 4.ODc), and strong biofilm production (4.ODc < OD). Mix = R1 + R2 + R3 + R4; OD = optical density.

Isolates from dairy industry products that showed biofilm formation activity were selected to determine the nucleotide sequence of nearly the entire length of the 16S rRNA gene, and results are reported in Table 1. R1 was *Klebsiella pneumoniae* 459, R2 was *Staphylococcus haemolyticus* M2, R3 was *Pseudomonas aeruginosa* RMR101, and R4 was *Aeromonas caviae* 8LM. The assessment of biofilm biomass production permitted to classify one as strong biofilm producer (R3), two as moderate biofilm producers (R1-R2), and one as weak producer (R4) (Figure 1). Because all four strains came from the same area after common disinfection procedures, we tested the ability to form biofilm together and we found that they were strongly biofilm producers as shown in Figure 1.

Determination of MIC and MBC values of melittin against Gram-positive and Gram-negative environmental isolates (R1–R4) are summarized in Table 2. Melittin exhibited a broad spectrum of antibacterial activity against both Gram-positive (MIC values 1–5 *µ*g/mL) and Gram-negative bacteria (MIC values between 50 and 100 *µ*g/mL). In the case of *P. aeruginosa*, melittin showed only bacteriostatic activity at considered concentrations (up to 100 *µ*g/mL).

**Figure 2:**
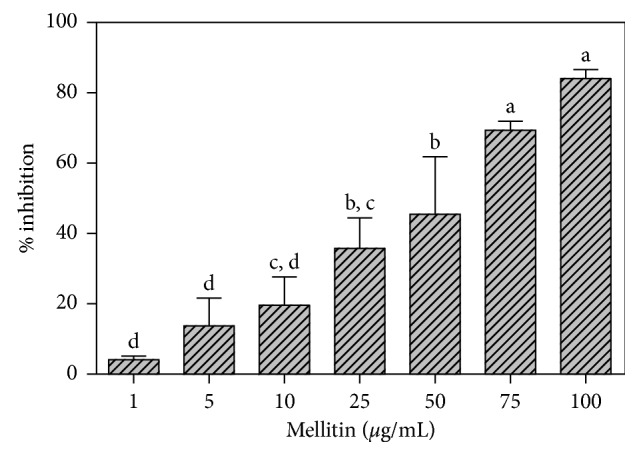
Effect of melittin on the inhibition of polymicrobial biofilm; data with different letters (a–d) are significantly different (Tukey's, *p* < 0.05).

Bacteria forming biofilms may be up to 1000 times more resistant to antimicrobial agents than those in a planktonic state. To evaluate the efficacy of melittin on the biofilm formation, either MIC or sub-MIC concentrations were used. Significant to moderate biofilm, reducing activity was observed for concentrations lower than these found for MIC. Melittin showed a dose-dependent inhibition of biofilm development according to the following equation: *Y* = −10 + 42 log *X* (*X* = inhibition concentration; *Y* = % inhibition; *R* ^2^ = 0.86; MSE = 142) (Figure 2). At the concentration of 5 *µ*g/ml, the inhibition reached 15%, while we have a 50% inhibition of PMB at 50 *µ*g/ml. In general, the inhibitory effect of melittin increased with increasing concentrations and the effect seemed to be dose-dependent.

**Figure 3:**
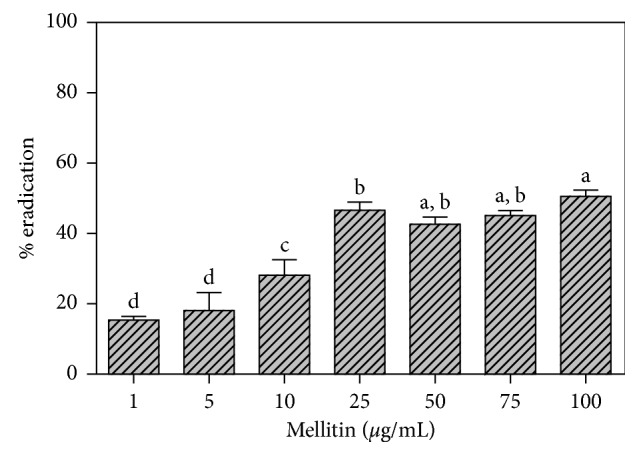
Action of melittin on eradication of polymicrobial biofilm; data with different letters (a–d) are significantly different (Tukey's, *p* < 0.05).

To corroborate the antibiofilm data, a colorimetric assay was performed to quantify biofilms remaining on surfaces after treatment. Results normalized to untreated controls (Figure 3) evidenced a dose-dependent percentage of eradication according to the following equation: *Y* = 9 + 19 log *X* (*X* = inhibition concentration; *Y* = % inhibition; *R* ^2^ = 0.89; MSE = 21). When we used the same concentrations to determine the ability of melittin to disperse mature biofilms, a significantly reduced biofilm mass at sub-MIC and MIC concentrations was noticed as shown in Figure 3. For example, the concentrations at 10 and 25 *µ*g/ml could disperse the preformed biofilms by 30% and 45%, respectively, showing an interesting better work of melittin on preformed biofilm at concentrations lower than for inhibition.

In addition, Pearson correlation analysis revealed as shown in Table 3 a positive and significant correlation between inhibition and eradication of single and mixed biofilms.

**Figure 4:**
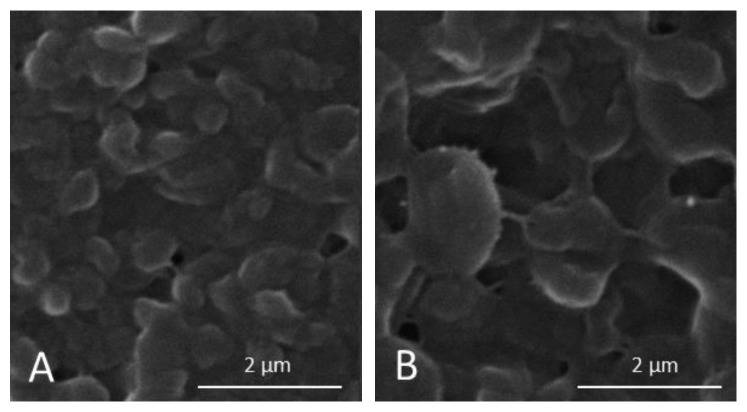
Morphology of mixed biofilm. The morphology of bacteria in the mixed biofilms visualized using the scanning electron microscope. Scale bar, 2 *µ*m. Panel A corresponds to 48 h mixed biofilms and panel B corresponds to treated 48 h mixed biofilms. At least three random fields were observed and analyzed, from three independent experiments.

SEM images after 48 h showed that melittin exposure had a great influence on the morphology and structure of mixed biofilms influencing both Gram-positive and Gram-negative bacteria. Melittin showed a reduction in biofilm formation and a variability in the thickness, with scattered damaged or dead cells, (Figure 4) confirming the data obtained by crystal violet and XTT assays.

Three main ways can be used to control biofilm formation: reduction of the planktonic cells before biofilm formation, initial prevention of cell adhesion to surfaces, and removal of mature biofilms.

It has been demonstrated in a lot of studies that peptides or antibiotics were able to inhibit the initial biofilm attachment (58–62%) at sub-MIC values. Probably, these molecules interact with bacterial adhesins that mediates the intercellular adhesion of bacteria to the surfaces [11]. Anyway, inhibition of biofilm formation in early critical stages is more applicable than inhibiting mature biofilm. The antibiofilm activities of AMPs are not completely understood, and there are a small number of studies that have marked possible explanations including matrix disruption, binding of DNA, and altering the expression of biofilm-related genes, such as the production of pili, quorum sensing systems, and flagella assembly, and their dual capacity to act on both the cytoplasmic membrane and intracellular targets [13, 23]. Several investigations have demonstrated the antibacterial activity of melittin [41]. Our results agree with Picoli et al. [25], which determined a higher sensitivity of Gram-positive microorganisms to melittin compared to the Gram-negative ones mainly due to their structural differences. Melittin can penetrate the peptidoglycan layer and reach the Gram-positive cell membrane more easily than in Gram-negative cells that have their membrane protected by a layer of lipopolysaccharides.

The industrial equipment used in cheese making and processing is a source of contamination, and the ability of microorganisms to adhere to the surfaces of them increases their potential contamination.

Biofilm formation is a process involving three stages: (i) primary adhesion to surfaces; (ii) accumulation of multilayered cells; and (iii) detachment. Most infections are caused by one single pathogen; however, polymicrobial infections have attracted more attentions because PMBs are the dominant form in nature. Synergistic, mutualistic, and antagonistic interactions that occur between microorganisms can collaborate to the growth of PMB communities, resulting in a stronger biofilm formation with increased antimicrobial tolerance. In food industry, PMBs are an important source of contamination for food and equipment, and thus a major cause of foodborne disease outbreaks.

Results demonstrated that melittin not only can inhibit early formation of PMBs in industrial equipment in a concentration-dependent way but also could easily penetrate the biofilm matrix preformed and reduce cell populations at sub-MIC concentrations.

Our data support the finding that melittin is more active against Gram-positive than Gram-negative bacteria, and this is due to the different cell wall and membrane structure in both types of bacteria so that melittin can penetrate the peptidoglycan envelope and reach the cell membrane of Gram-positive easier than for Gram-negative that are protected also by the lipopolysaccharide envelope [42].

These results suggest that melittin could be used in the dairy food industry to control PMB formation, as an alternative or in combination with conventional sanitizers.

## 4. Conclusions

Results suggested that PMBs inhibition and eradication from diary isolates could be treated by melittin after usual cleaning procedures to further remove any form of resistance. Anyway, PMB cannot be always easily and effectively removed due to their great level of resistance and diversity in the mix-of-species composition. Further studies are required to understand the mechanisms of action and the better association between active antimicrobial molecules and PMB mix-of-species.

## Figures and Tables

**Table 1:** The nucleotide sequences of the 16s rRna gene of the isolates were compared with the known 16s rRna gene nucleotide sequences available in the ddBJ/genbank/emBl databases using the NCBI sequence database. Accession numbers for the available 16s rrna gene sequences used are given in parentheses after the species and strain names.

Sample	Strains
R1	*Klebsiella pneumoniae* strain 459 chromosome, complete genome (KM609195.1 MF321768.1)
R2	*Staphylococcus haemolyticus* strain M2_0m_PrM_10 16S ribosomal RNA gene, partial sequence (KY742479.1 KX946163.1)
R3	*Pseudomonas aeruginosa* strain RMR101 16S ribosomal RNA gene, partial sequence (CP018306.1 MG396990.1)
R4	*Aeromonas caviae* strain 8LM chromosome, complete genome (CP024198.1 KX980472.1)
Mix	R1 + R2 + R3 + R4

**Table 2:** Minimum inhibitory concentration (MIC) and minimum bactericidal concentration (MBC) of melittin against environmental isolates.

	MIC (*µ*g/mL)	MBC (*µ*g/mL)	MIC/MBC ratio	
*K. pneumoniae*	50	100	2	Bactericide
*S. haemoliticus*	1	3	3	Bactericide
*P. aeruginosa*	100	>100	—	Bacteriostatic
*A. cavia*	25	50	2	Bactericide

**Table 3:** Pearson's correlation between species occurrence and contact time.

	24 h	48 h	*K. pneumoniae*	*S. haemoliticus*	*P. aeruginosa*	*A. cavia*
24 h		**−0.995**	**0.925**	0.593	**0.974**	**0.907**
48 h	**−0.995**		**−0.951**	−0.557	**−0.971**	**−0.901**
*K. pneumoniae*	**0.925**	**−0.951**		0.535	**0.896**	**0.804**
*S. haemoliticus*	0.593	−0.557	0.535		0.560	0.682
*P. aeruginosa*	**0.974**	**−0.971**	**0.896**	0.560		**0.930**
*A. cavia*	**0.907**	**−0.901**	**0.804**	0.682	**0.930**	

Values in bold are statistically significant (*p*=0.05).

## Data Availability

The data used to support the findings of this study are included within the article.
